# Medial Swivel Injury: Misdiagnosis in Four Patients Over Three Months

**DOI:** 10.7759/cureus.114024

**Published:** 2026-08-05

**Authors:** Alireza Mousavian, Yaghoob Keikha

**Affiliations:** 1 Orthopedic Research Center, Mashhad University of Medical Sciences, Mashhad, IRN; 2 Orthopedic Surgery Department, Mashhad University of Medical Sciences, Mashhad, IRN

**Keywords:** delayed diagnosis, delayed treatment, misdiagnosis, swivel injury, talonavicular dislocation

## Abstract

Medial swivel injury is a rare injury among midfoot fracture-dislocations, typically caused by direct and high-energy force applied to the midfoot region. However, it can also develop after minor trauma, like a foot or ankle sprain sustained during sports or walking. Swivel type of injury refers to an isolated dislocation of the talonavicular joint (TNJ) with the calcaneum getting swiveled beneath the talus (i.e., subtalar subluxation), with intact introsseous ligament and intact calcaneocuboid joint. However, in our experience, in practice, it is more commonly associated with fracture-dislocation in the talonavicular area rather than being merely a dislocation or ligamentous injury. Diagnosis of this injury requires meticulous attention during the initial physical examination and appropriate radiological evaluation. Without sufficient care, the diagnosis can easily be missed, leading to a delay in treatment. The clinical literature on swivel fracture dislocation is limited. Following the referral of four cases with missed diagnosis and inadequate treatment over a three‑month period, we present this article to emphasize the significance of this injury and to review the appropriate diagnostic and therapeutic approaches.

## Introduction

Midtarsal joint dislocation, which is composed of talonavicular and calcaneocuboid articulations, is a quite rare injury of the foot and ankle. This is attributed to its strong periarticular ligamentous support at this joint. This injury mostly affects middle-aged men. It is most commonly secondary to a major trauma and is generally associated with a fracture rather than an isolated pure dislocation [1-4]. A subtype pattern of these injuries is the swivel dislocation at the talonavicular joint (TNJ). A medial or lateral force to the forefoot can lead to a swivel dislocation at the talonavicular joint without disruption of the subtalar joint [5-7]. Sometimes, it can develop after minor trauma, like a foot or ankle sprain sustained during sports or walking [8]. Medial dislocation is the most frequent type, accounting for approximately 80% of cases reported in the literature [9,10]. Pain and swelling around the foot are the usual complaints, but patients may present with acute skin compromise. Patients usually present immediately to the emergency room (ER) after injury, but late presentation has also been reported. X-rays and computed tomography (CT) scans are usually required for making a diagnosis. Due to the rare incidence of this injury, sometimes, X-rays might be read as normal [11-13]. Midtarsal injuries, especially pure dislocations and subluxations, are commonly missed injuries, often misdiagnosed as sprains, due to the occult nature of radiographic findings. Studies suggest that 41% of these injuries are missed during initial assessment due to difficulties in diagnosing purely through radiographs [14,15].

The initial diagnosis is based on physical examination and radiography. On the anteroposterior (AP) foot view, the navicular bone is seen positioned either medial or lateral to the talar head. A computed tomography (CT) scan is necessary to obtain a diagnosis and formulate a surgical plan. Various treatment methods have been used for this injury; closed reduction can only be applied if the injury is purely ligamentous. The management is challenging, and the treatment is fraught with a high rate of disability [9].

## Case presentation

Case 1

**Figure 1:**
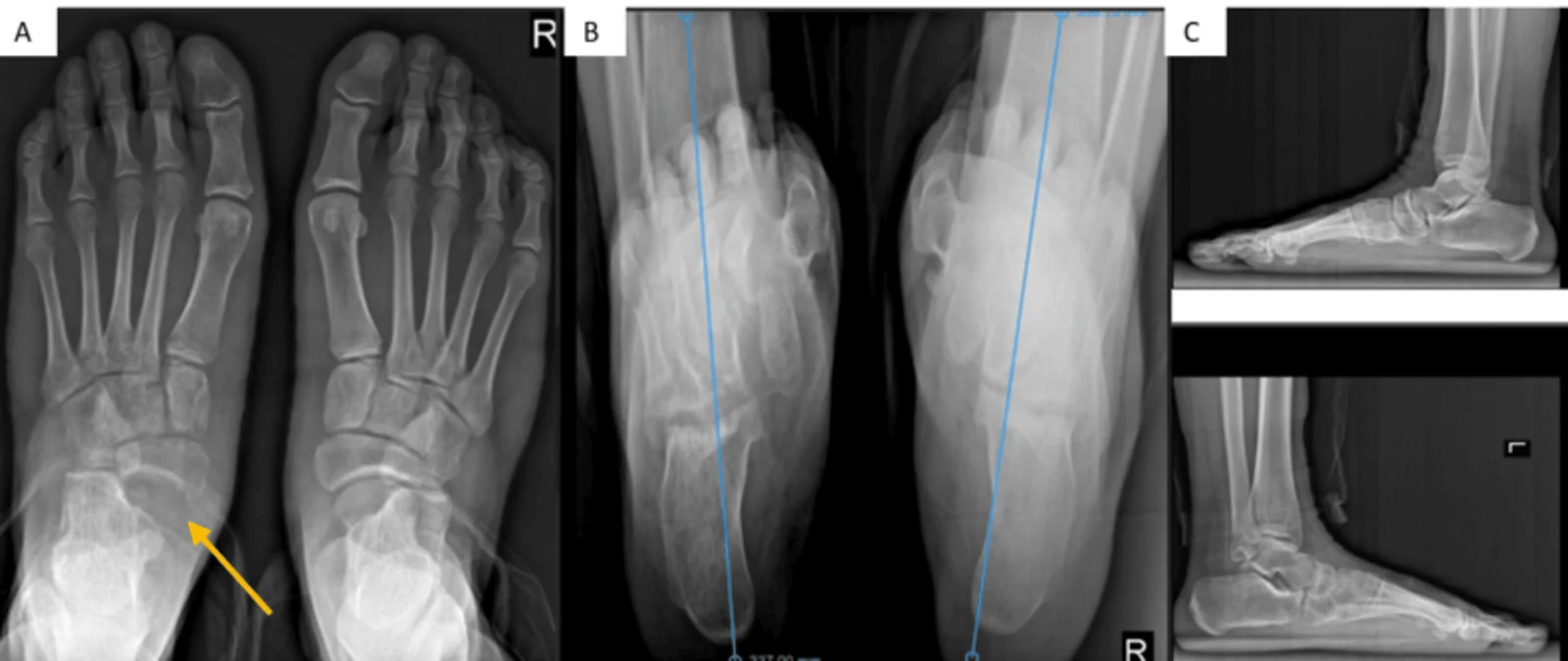
Preoperative radiographs taken three months after the injury upon referral to our clinic (A) Anteroposterior view of the foot (arrow showing left foot medial swivel dislocation), (B) Saltzman view, and (C) lateral view

**Figure 2:**
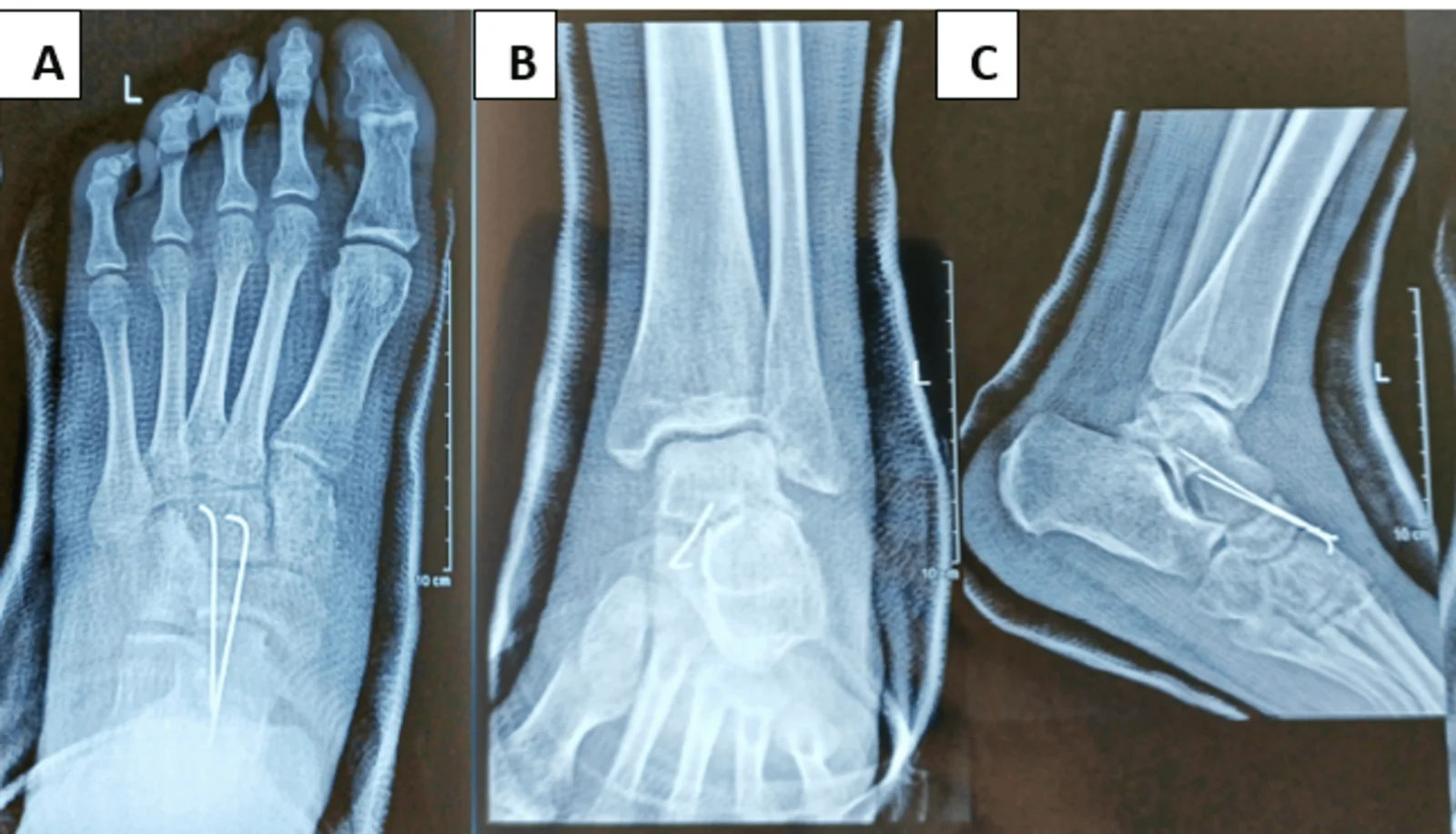
Postoperative radiographs two weeks after surgery (A and B) Anteroposterior view and (C) lateral view

A 23-year-old man twisted his left foot while playing basketball. The emergency department assured him that there was no problem other than a simple sprain and casted his foot. After one month, he was able to walk with some discomfort and a little varus, which could hardly be diagnosed in physical examination. After three months, he came to my foot clinic. His foot appeared fine, without significant swelling or deformity. Five standard hindfoot views, including two ankle and foot views and a Saltzman view, were taken (Figure 1). We were able to diagnose the medial swivel talonavicular fracture dislocation, which had been neglected by the patient and providers, probably because of minor symptoms and signs. The procedure was done in the OR under general anesthesia with the patient in supine position. A mid-dorsal incision was made in line with the lateral cuneiform. The navicular fossa was filled with incarcerated soft tissues and scars, as well as some remnants of cancellous bone and cartilage from the talar head. The head was stuck laterally to the navicular bone without any motion. Gentle mobilization with a Penfield dissector was done. Cartilage assessment showed normal-appearing navicular and remaining talar head cartilage. Navicular fossa was cleaned. A second lateral sinus tarsi approach was carried out to ensure mobilization was possible without excessive soft tissue contracture. A pin distractor was applied on the talar head and navicular, and distraction was combined with de-twisting the talar head as much as possible. Direct lateral window confirmed complete subtalar dislocation, as well as 80% talonavicular reduction in direct dorsal view and fluoroscopy. Reduction was completely unstable and hence was stabilized with two smooth 1.5 mm pins in the talonavicular joint (Figure 2).

**Figure 3:**
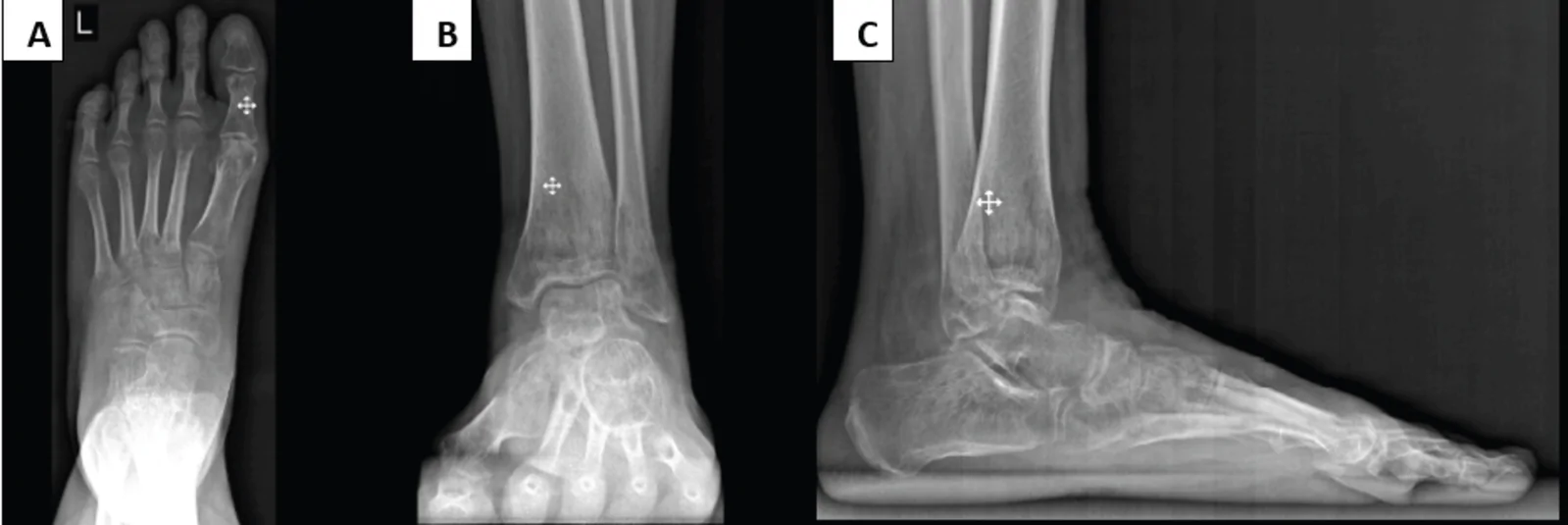
Two months after surgery and 10 days after pin removal (A and B) Anteroposterior view and (C) lateral view

In this case, we used a smooth pin for fixation due to the size of the bone, intact joint cartilage, and absence of fracture. After two months of surgery and pin removal, there is no pain and no varus deformity (Figure 3).

Case 2

**Figure 4:**
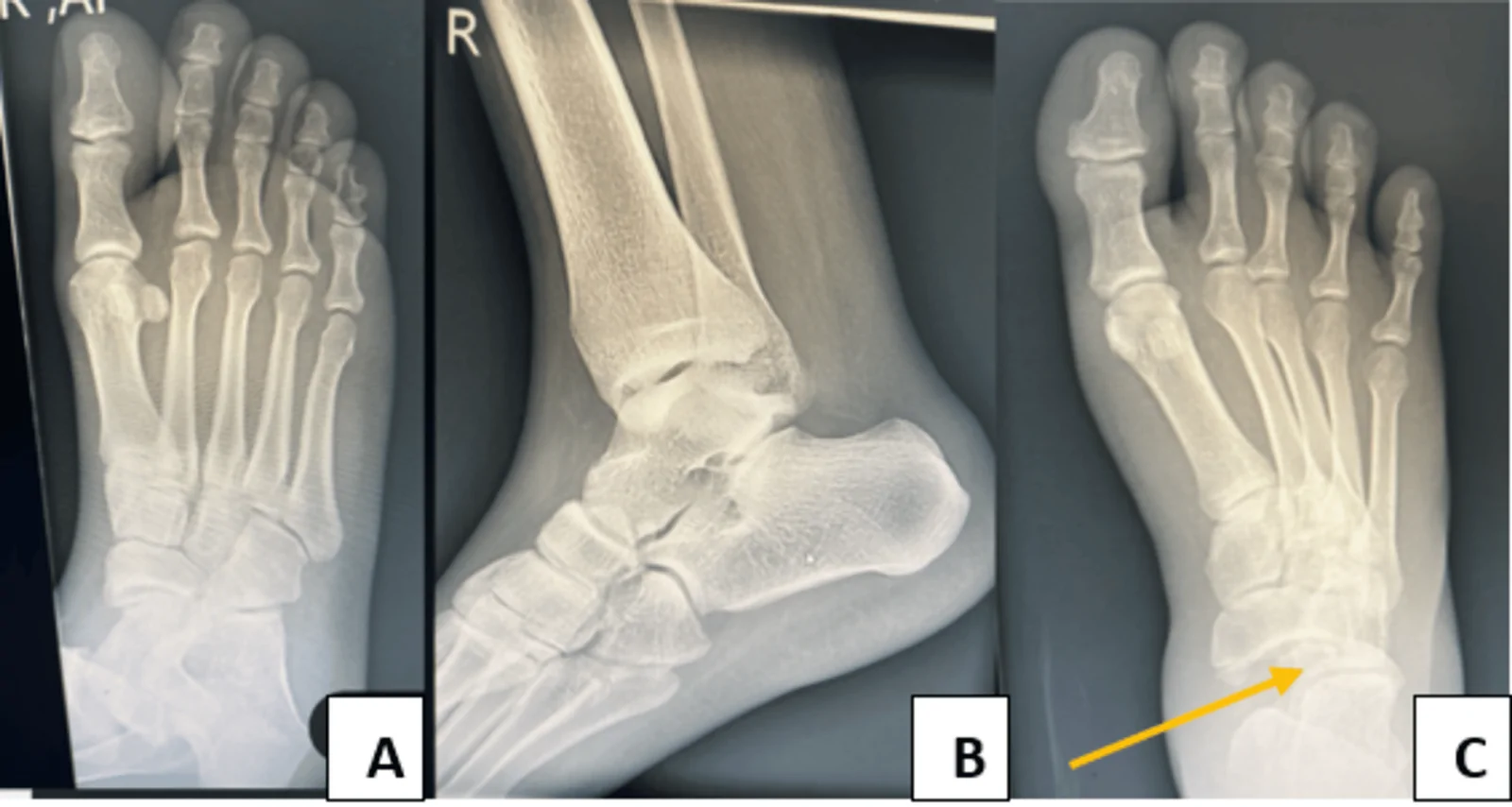
Preoperative radiographs taken three weeks after injury (A) Anteroposterior view, (B) lateral view, and (C) oblique view (arrow indicating right foot medial swivel injury)

**Figure 5:**
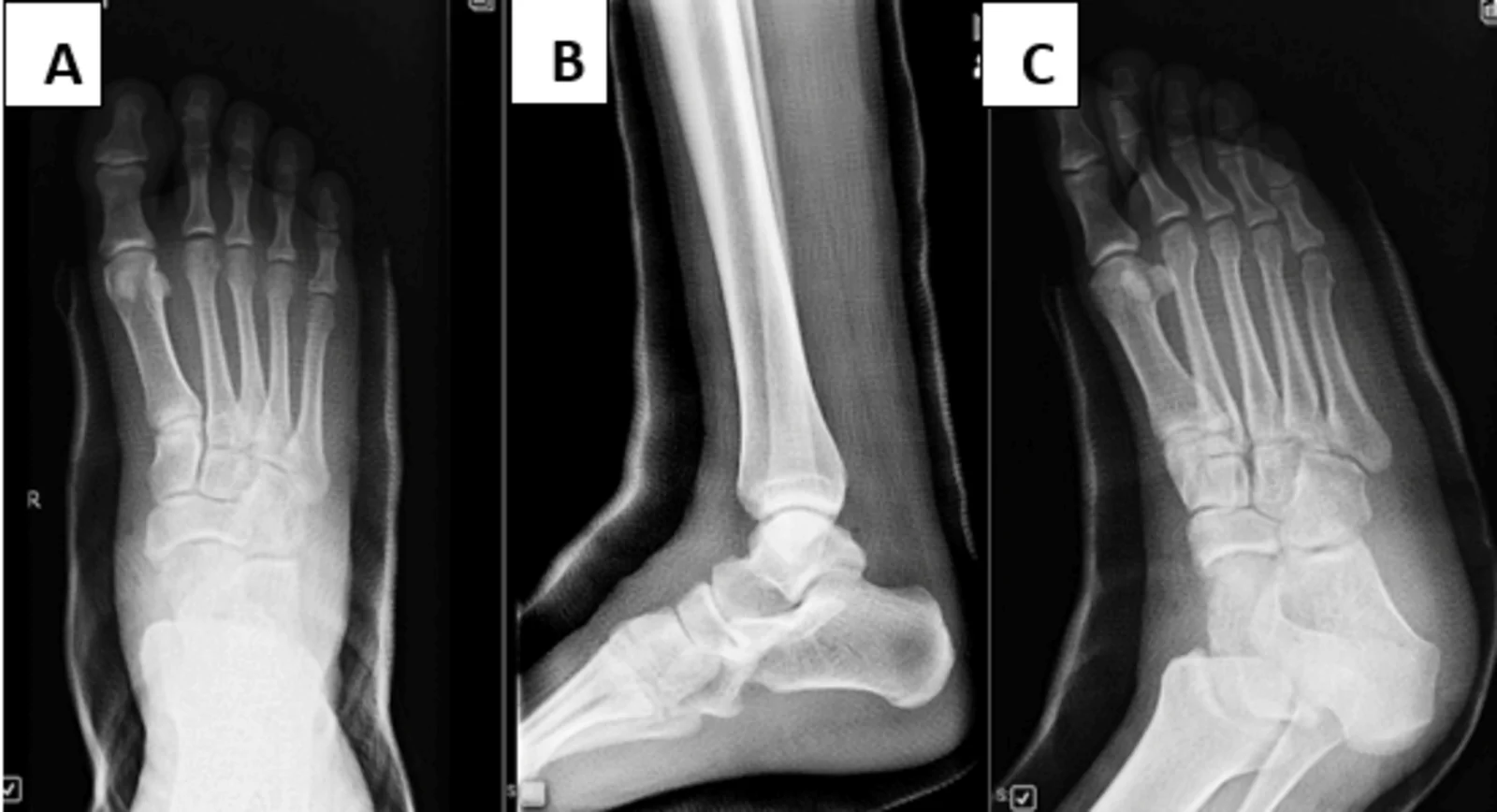
Postoperative radiographs one month after surgery showing good reduction and fixation (A) Anteroposterior view, (B) lateral view, and (C) oblique view of the foot

**Figure 6:**
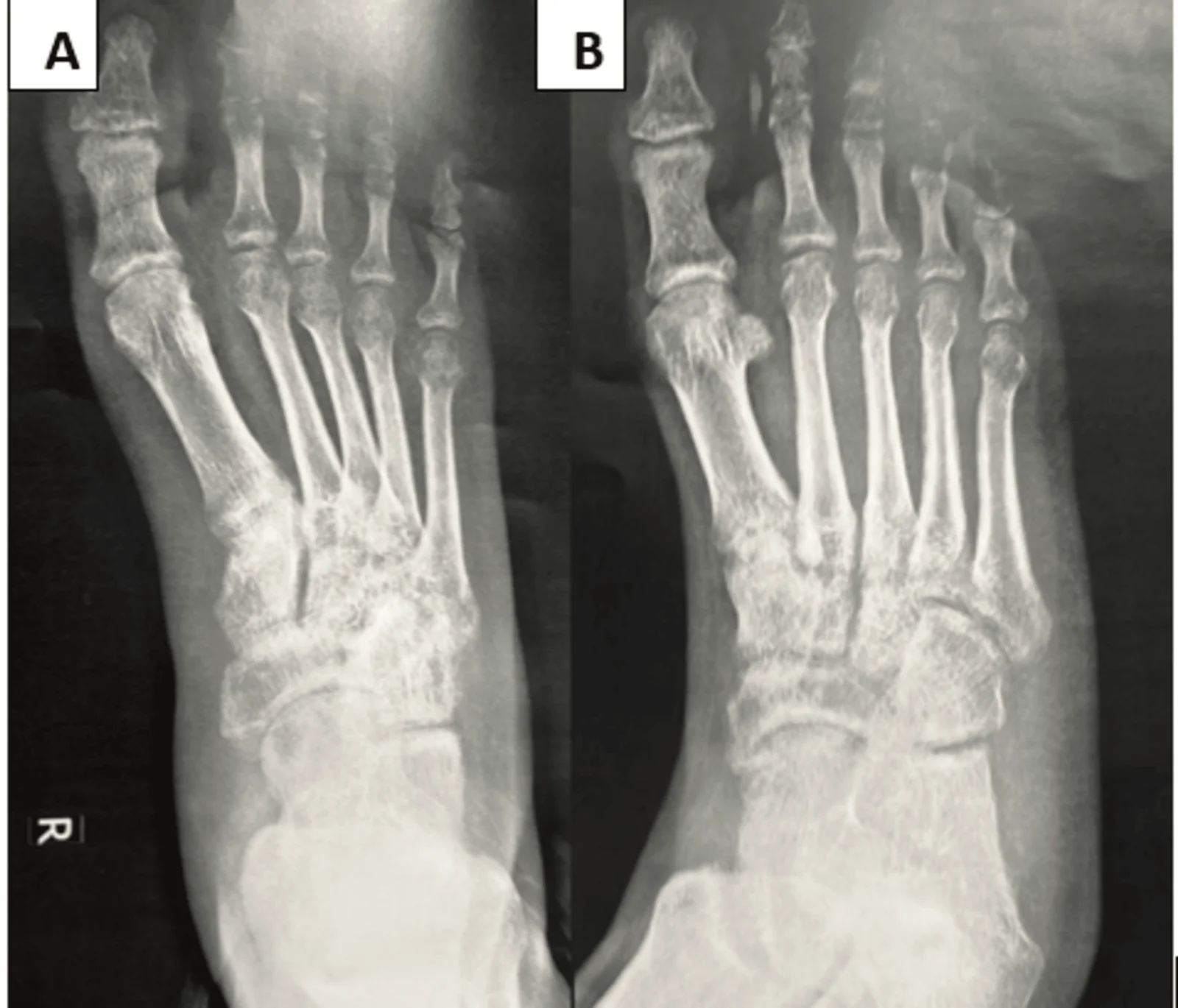
Two months after surgery (A) Anteroposterior view and (B) oblique view

Twenty days before coming to us, a 22-year-old man presented to another medical center due to an ankle sprain while walking on an uneven surface. He presented to us following referral to a hospital in another city and evaluation by an orthopedic colleague. X-rays were performed, and he had been treated with cast immobilization. However, at three weeks post-injury, because there was no reduction in swelling or pain and no symptomatic improvement, he was referred to our clinic. On examination, pain, swelling, inability to bear weight during ambulation, and varus deformity were evident. On radiography, fracture-dislocation of the talar head was evident (right foot medial swivel injury) (Figure 4). In the operating room, with the patient under general anesthesia and in supine position, a dorsomedial incision was initially made over the Chopart joint. After the release of the superficial layer of the deltoid ligament, reduction was attempted, but due to the time elapsed since injury, it was irreducible. However, there was no newly formed soft tissue within the joint space requiring excision. Subsequently, a lateral approach through the sinus tarsi was performed for further release, and using a distractor, the talonavicular joint was distracted and successfully reduced. For the talar head fracture, following correction of the impaction, a provisional pin was placed first, and then, based on the fragment size, fixation was achieved using one absorbable pin. Finally, bone allograft was placed at the site of impaction to prevent future collapse. After checking with fluoroscopy, the reduction and reconstruction of the articular surface were found to be satisfactory (Figure 5). The superficial layer of the deltoid ligament was repaired. In this case, we used an absorbable pin for fixation due to a small interarticular talus fragment. After two months of surgery, the patient has no pain, swelling, or deformity in the right foot. The patient's follow-up care is ongoing (Figure 6).

Case 3

**Figure 7:**
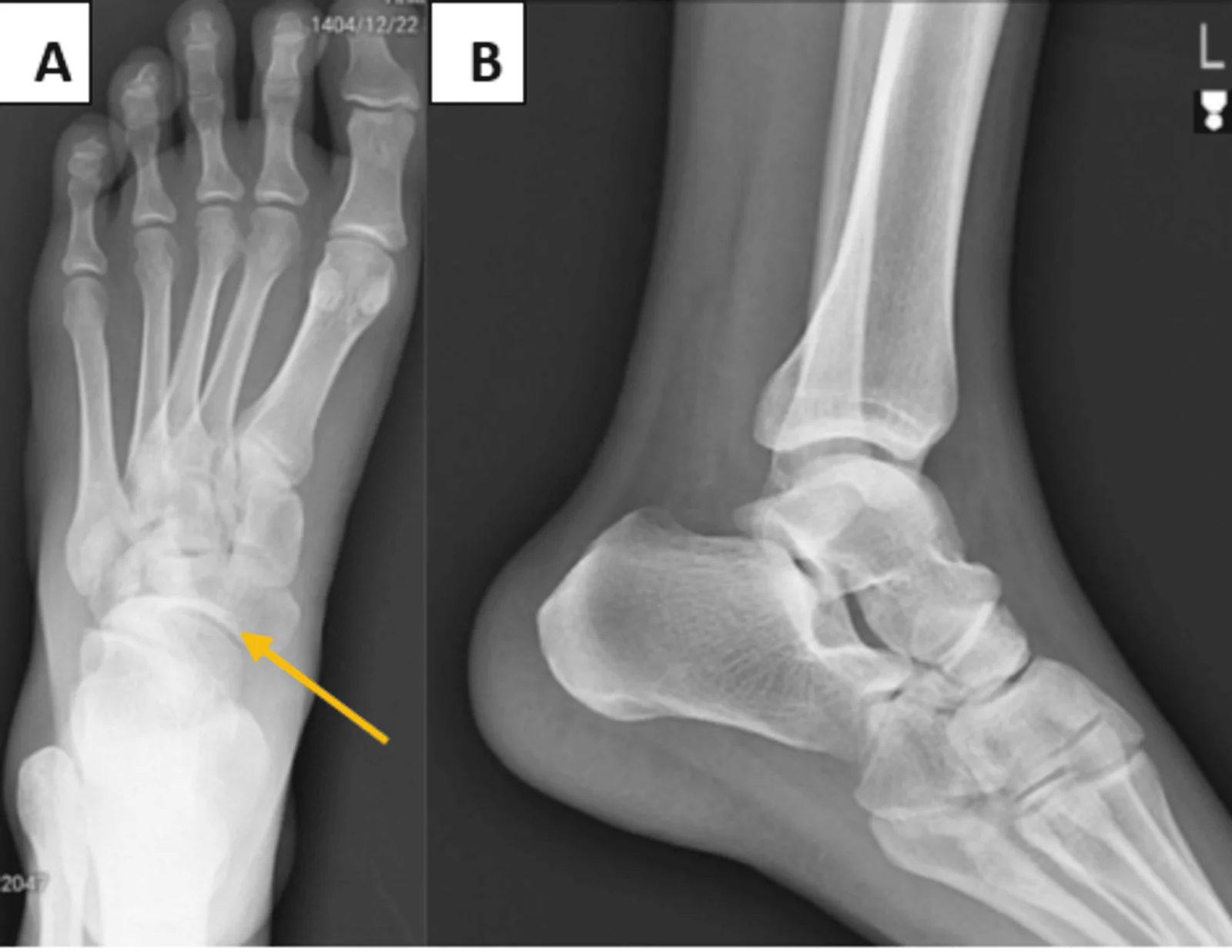
Preoperative radiographs taken 16 days after injury (A) Anteroposterior view and (B) lateral view (arrow showing left foot medial swivel injury)

**Figure 8:**
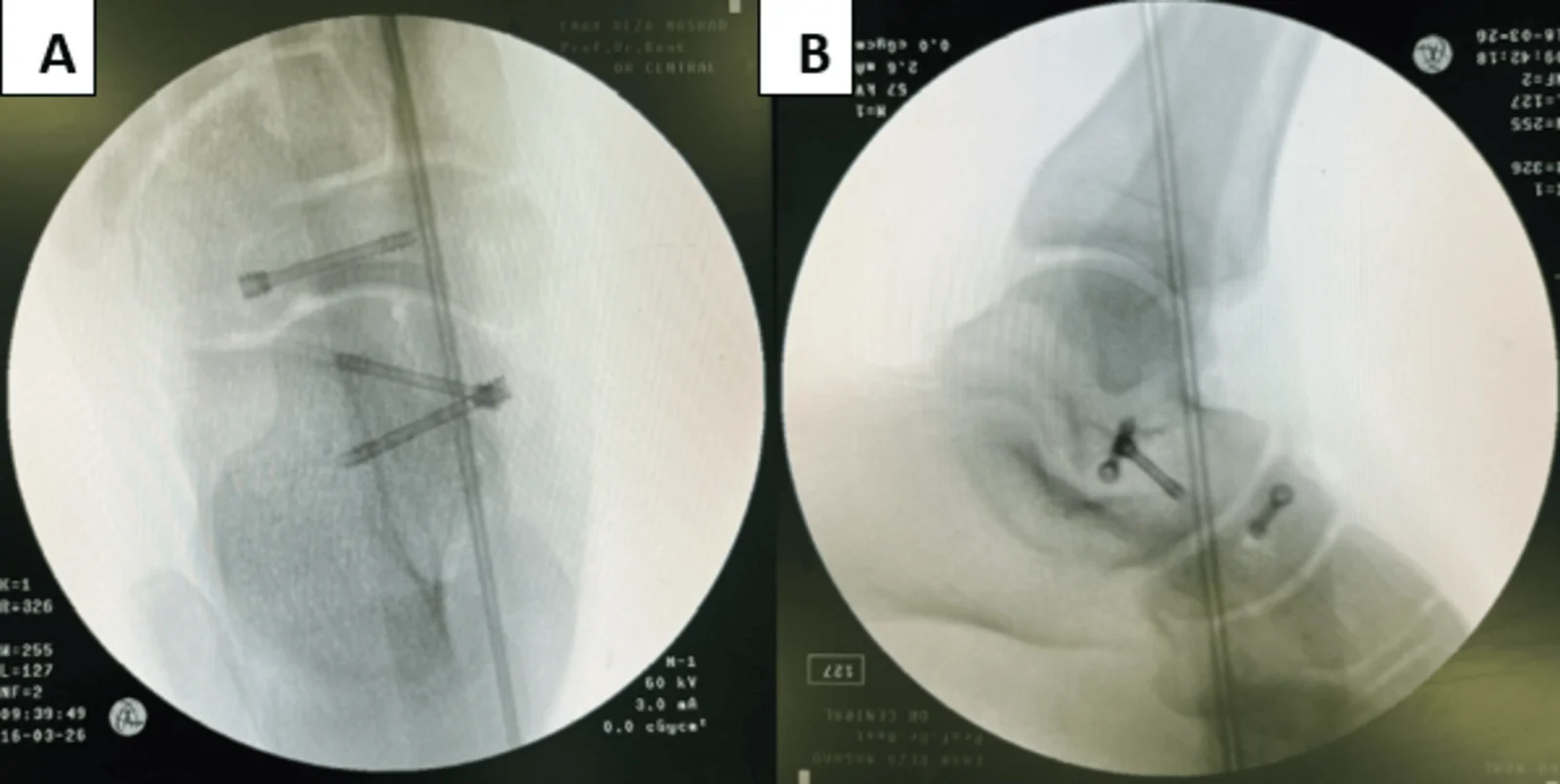
Immediate postoperative radiographs (A) Anteroposterior view and (B) lateral view showing good reduction and fixation

**Figure 9:**
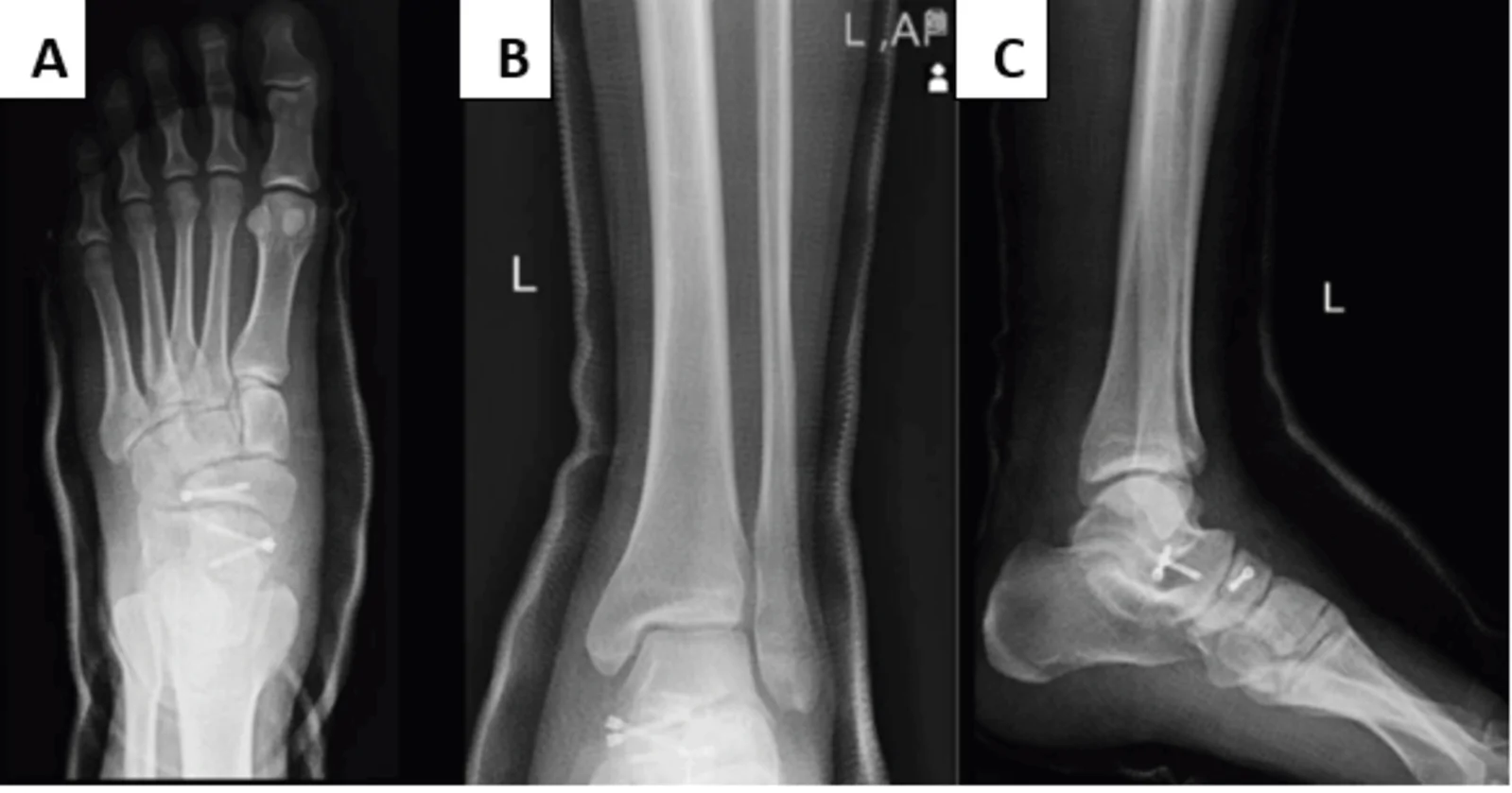
One month after surgery (A and B) Anteroposterior view and (C) lateral view in casting

A 34-year-old man presented to another medical center 16 days before coming to our center due to a foot sprain while playing football. He had been treated with taping/splinting, but because there was no decrease in pain and swelling after two weeks, the patient was referred to our clinic. On examination, pain and swelling were evident in the left foot, and the foot was seen in an equinovarus position. The patient was unable to bear weight on this limb. The patient underwent radiographs of the foot and ankle, followed by a CT scan, which showed a medial talonavicular fracture-dislocation, cuboid fracture, and impaction fracture involving the lateral and inferior portions of the talar head (Figure 7). In the operating room, under general anesthesia in supine position, the patient was explored via a dorsomedial foot incision. Findings included a fracture of the navicular bone and impaction fracture of the head of the talus. There was no newly formed soft tissue within the joint space requiring excision. With the help of a distractor, reduction of the talonavicular joint was done gently; then, fixation of fractures of the navicular and the head of the talus was carried out using headless screws under the guidance of fluoroscopy, which was successful (Figure 8). In this case, we used a headless screw to achieve stable and good fixation in navicular and talus fractures. At one-month follow-up radiographs, reduction and fixation showed good results (Figure 9). The patient continues to be followed up.

Case 4

**Figure 10:**
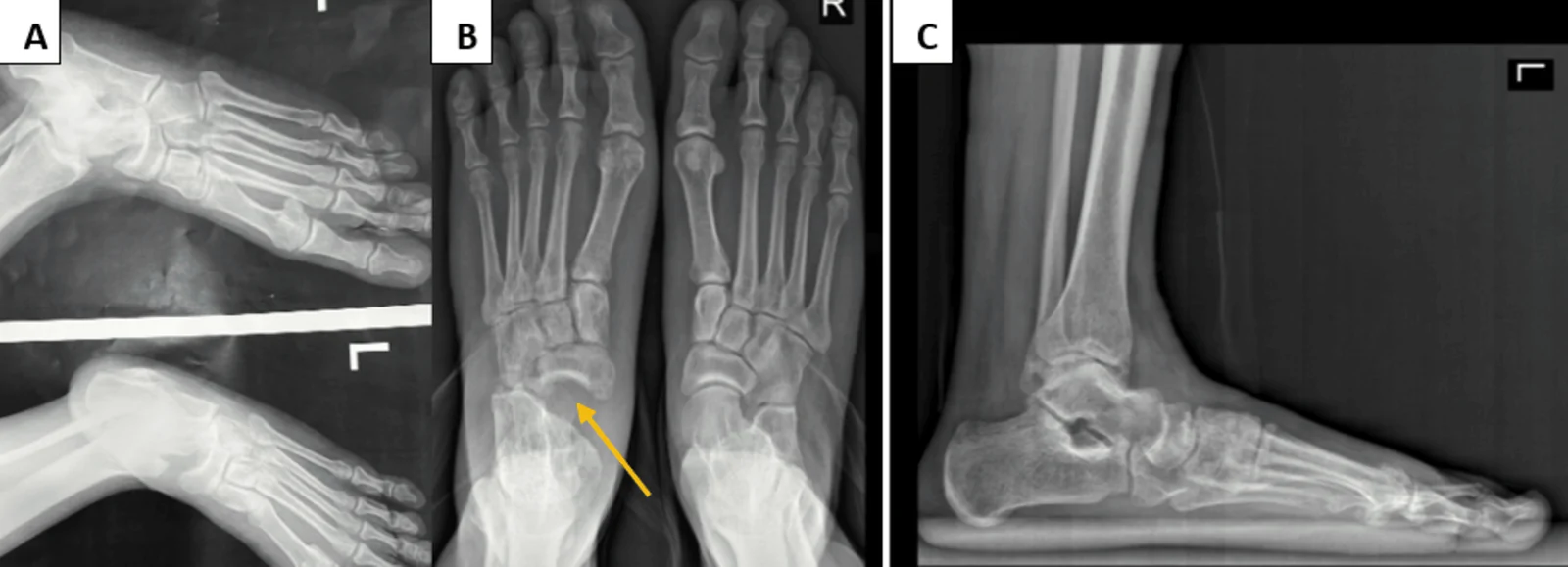
Preoperative radiographs taken 40 days after injury (A) Oblique view, (B) anteroposterior view, and (C) lateral view (arrow showing left foot medial swivel injury)

**Figure 11:**
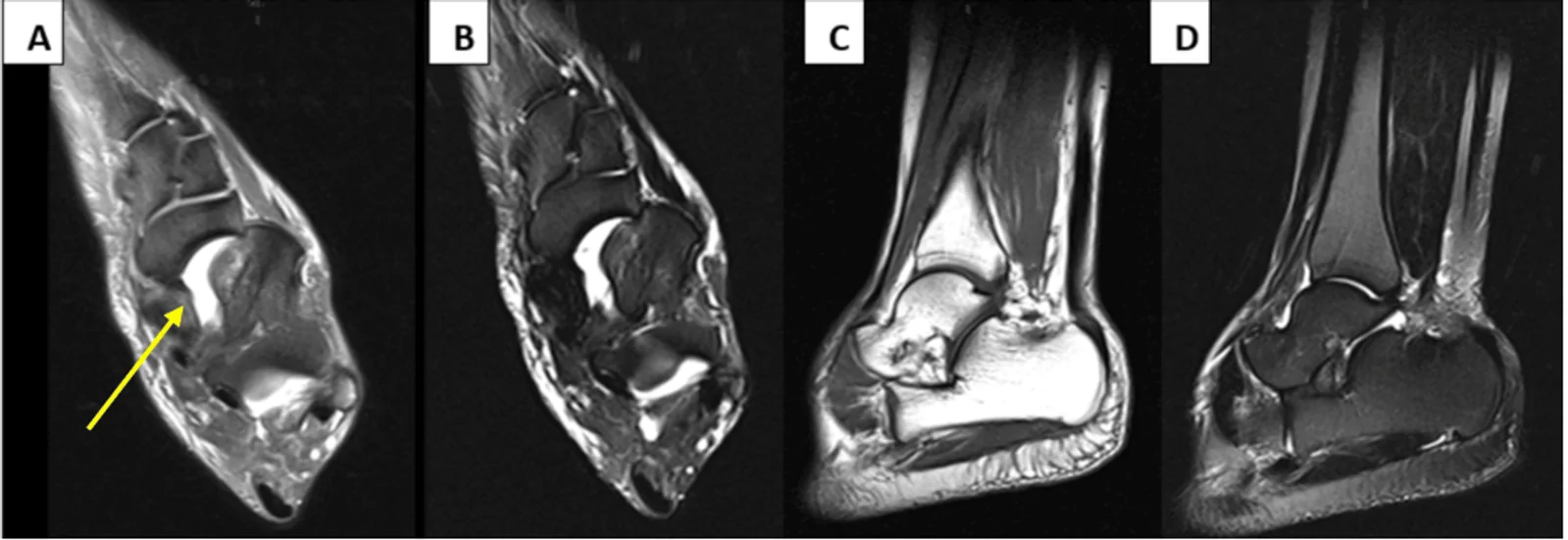
Preoperative MRI images (A-D) 30 days after injury showing left foot medial swivel injury Arrow showing medial talonavicular dislocation MRI: magnetic resonance imaging

**Figure 12:**
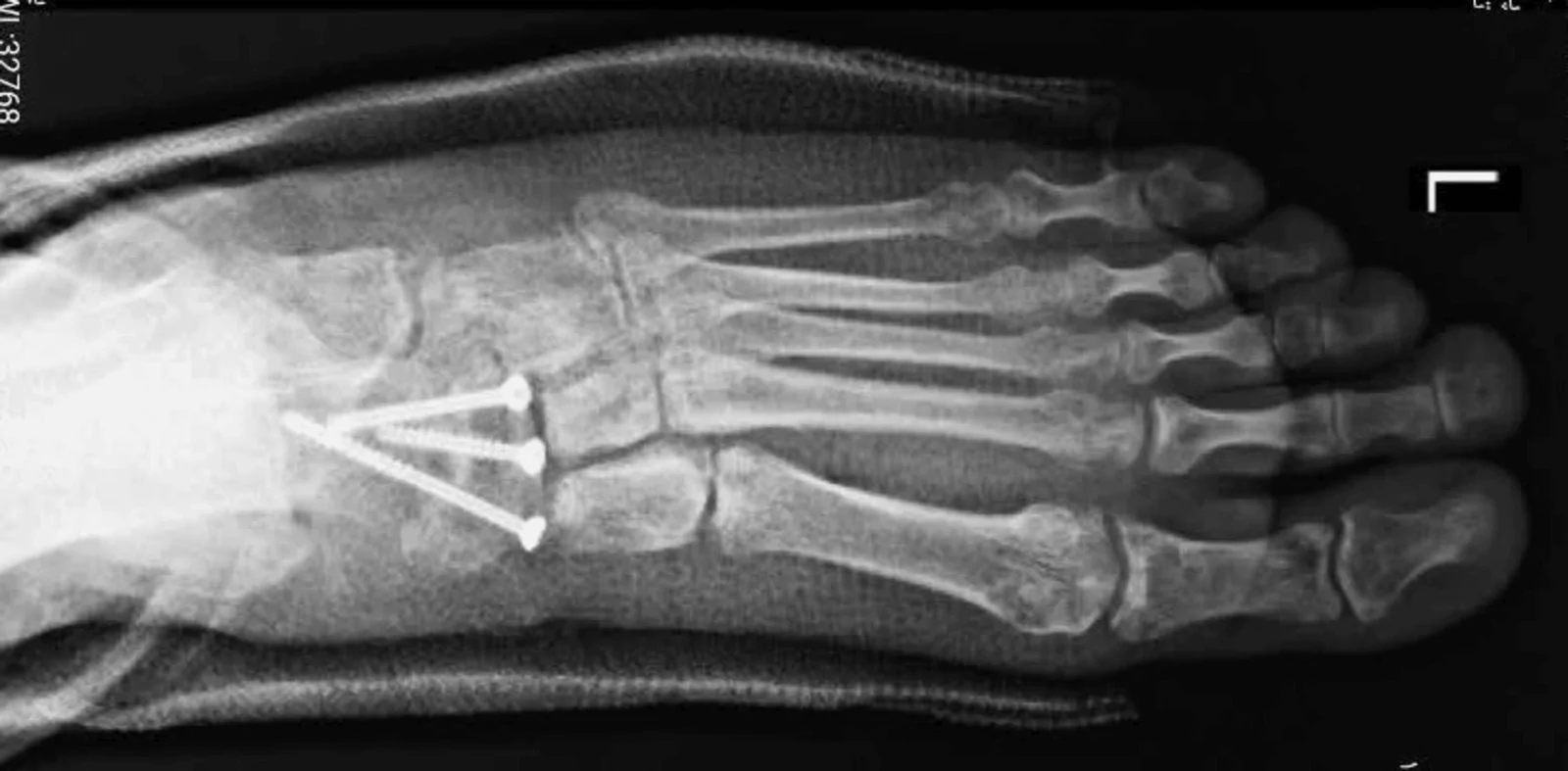
Postoperative radiograph one month after surgery

Following a left ankle sprain, a 38-year-old man presented to another medical center three months before coming to our center and was treated with cast immobilization for 40 days. Due to lack of improvement in pain and swelling and inability to bear weight on the affected limb, he presented to our center. On examination, mild varus deformity, in addition to the abovementioned symptoms, was notable. We performed radiographs of the foot and ankle for the patient, in which a neglected medial swivel injury in the left foot was observed. A notable point concerning this patient is that, in spite of evaluation by both an emergency physician and an orthopedic specialist, and despite the performance of radiography, CT scanning, and even MRI, neither a correct diagnosis nor appropriate treatment was rendered (Figures 10, 11). Although the patient was not operated on at our clinic, our follow-up of him and the foot and ankle surgeon colleague who treated him shows that he had undergone open reduction and internal fixation with cannulated screws. As shown in the postoperative radiograph, three 4-mm cannulated screws have been placed from the navicular bone toward the head of the talus. In a follow-up contact we had with him two months after the surgery, he expressed satisfaction with the improvement of symptoms such as pain and swelling, as well as the improvement of the apparent foot deformity, and he sent us a postoperative radiograph (Figure 12). The patient reported using axillary crutches for walking and had started partial weight-bearing two months after the surgery.

Table 1 provides an overview of the four presented cases.

**Table 1:** Summary of presented cases DX: dislocation, ORIF: open reduction and internal fixation, FX: fracture

Case	Age	Side	Injury to diagnosis time interval	Mechanism of injury	Radiographic findings	Treatment	Outcome
Case 1	23	Left	3 months	Sprain	Medial talonavicular DX	ORIF	No pain
						Pin	No deformity
Case 2	22	Right	3 weeks	Sprain	Medial talonavicular DX and talus FX	ORIF	No pain
						Absorbable pin	No deformity
Case 3	34	Left	16 days	Sprain	Medial talonavicular DX, and navicular and talus FX	ORIF (headless screw)	No pain
							No deformity
Case 4	38	Left	40 days	Sprain	Medial talonavicular DX	ORIF (canulated screw)	No pain
							No deformity

## Discussion

A medial peritalar fracture-dislocation is not a common injury. This injury has the potential for misdiagnosis, delayed treatment, and the development of complications. The hallmarks of these injuries were described by Ricci et al. [16]. The best therapeutic approach for this injury remains controversial [17]. Dislocations of the midtarsal joint are a highly uncommon injury because of the strong periarticular ligamentous support at this joint. According to estimates, there are 3.6/100,000 cases of this injury yearly. The medial and lateral longitudinal columns of the midfoot are disrupted in TNJ dislocation, which often follows high-energy trauma. It is also believed that a greater disruption would be necessary to result in an isolated dislocation without navicular body fracture. The foot turns medially along the axis of the intact talocalcaneal ligament (which prevents subtalar dislocation) [18-20]. However, many case studies concluded that low-energy trauma, as in our cases, can also result in such an injury. It is rare to have an isolated talonavicular joint dislocation without concurrent talocalcaneal joint dislocations or bone fractures due to the strong ligamentous and bony framework supporting the midtarsal joints [21]. Up to 80% of TNJ dislocations are medial, and 17% are lateral [22]. Main and Jowett (1975) described such injuries based on the force applied as having five basic types: medial, longitudinal, lateral, plantar, and crush injuries. The injury patterns described by Main and Jowett have been an easy way to understand midtarsal dislocations. They included 71 patients in their study, 31 of whose diagnoses were delayed due to inadequate radiographs [14]. Neglect of these injuries has been reported in up to 41% of acute midtarsal dislocation, although Howie et al. reported that five out of seven patients in their study on midtarsal occult subluxation were initially missed [23]. In case of an acute injury, the recommended treatment for best outcomes is open reduction and internal fixation for both pure type and fracture dislocations [24]. Research articles do suggest that a higher index of suspicion is needed to diagnose such injuries, as neglect may lead to permanent disability [25].

**Figure 13:**
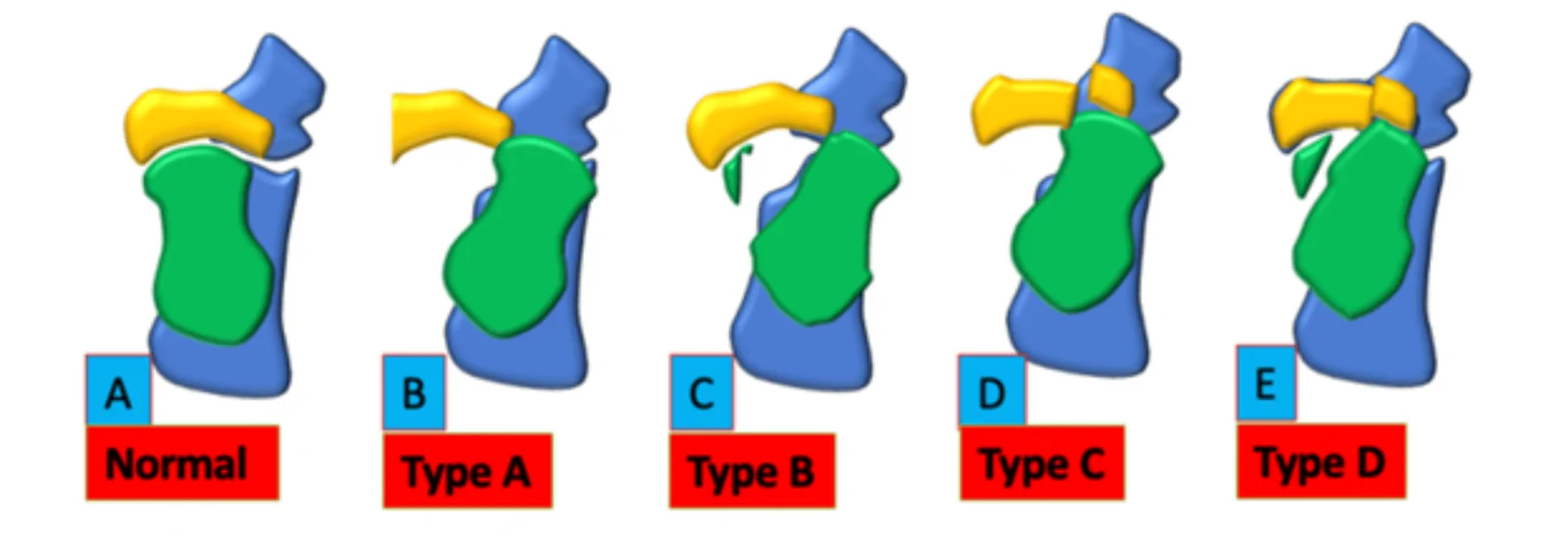
Patterns of acute talonavicular dislocation (A) Normal hindfoot, (B) pure swivel-type talonavicular medial dislocation, (C) talonavicular joint fracture dislocation with fracture in the medial side of the talar head, (D) talonavicular fracture dislocation with fracture in the lateral column of the navicular bone, and (E) talonavicular joint fracture dislocation with fracture in the medial side of the talar head and lateral navicular column

In continuation, we presented a definition of the pathogenesis and classification of this injury, summarizing the resources available to us along with our own experiences. Pathogenesis consists of two main conditions. First, the talonavicular joint is part of two complex joints: the first is subtalar + talonavicular, and the second is talonavicular + calcaneocuboid (Chopart joint). The first structure conjoins in sagittal foot motion, and the second structure forms a conjoined joint in supination-pronation motion. Second, talus biomechanics transfers forces from the ankle joint to the subtalar and talonavicular joints. Sometimes, talonavicular dislocation affects the ankle joint, which protects against failure of the subtalar joint. To summarize our own therapeutic experience regarding this injury and to provide an easier treatment approach for our orthopedic surgeon colleagues, and given the limited number of published articles on this topic, we present the patterns of acute talonavicular dislocation in Figure 13.

## Conclusions

The four cases presented in this study all sustained medial swivel injuries as fracture-dislocations due to low-energy trauma to the midfoot region, and all of them experienced a delay in diagnosis and subsequent delay in treatment due to failure of detection at the initial presentation. Failure to initially diagnose a medial swivel injury in the midfoot region can sometimes lead to permanent complications and the need for treatments such as joint fusion and progressive foot collapse. On the other hand, with early diagnosis and appropriate, timely treatment, patients can potentially achieve full functional recovery. Making an accurate diagnosis in this injury necessitates a comprehensive clinical examination, full radiographic imaging of the foot and ankle, and a CT scan.
